# Germline de novo variant F747S extends the phenotypic spectrum of *CACNA1D* Ca^2+^ channelopathies

**DOI:** 10.1093/hmg/ddac248

**Published:** 2022-10-08

**Authors:** Ferenc Török, Kamer Tezcan, Ludovica Filippini, Monica L Fernández-Quintero, Lucia Zanetti, Klaus R Liedl, Raphaela S Drexel, Jörg Striessnig, Nadine J Ortner

**Affiliations:** Department of Pharmacology and Toxicology, Center for Molecular Biosciences Innsbruck, University of Innsbruck, Innsbruck 6020, Austria; Department of Genetics, Kaiser Permanente, Sacramento, CA 95825, USA; Department of Pharmacology and Toxicology, Center for Molecular Biosciences Innsbruck, University of Innsbruck, Innsbruck 6020, Austria; Department of General, Inorganic and Theoretical Chemistry, Center for Molecular Biosciences Innsbruck, University of Innsbruck, Innsbruck 6020, Austria; Department of Pharmacology and Toxicology, Center for Molecular Biosciences Innsbruck, University of Innsbruck, Innsbruck 6020, Austria; Department of General, Inorganic and Theoretical Chemistry, Center for Molecular Biosciences Innsbruck, University of Innsbruck, Innsbruck 6020, Austria; Department of Pharmacology and Toxicology, Center for Molecular Biosciences Innsbruck, University of Innsbruck, Innsbruck 6020, Austria; Department of Pharmacology and Toxicology, Center for Molecular Biosciences Innsbruck, University of Innsbruck, Innsbruck 6020, Austria; Department of Pharmacology and Toxicology, Center for Molecular Biosciences Innsbruck, University of Innsbruck, Innsbruck 6020, Austria

## Abstract

Germline gain-of-function missense variants in the pore-forming Cav1.3 α1-subunit (*CACNA1D* gene) confer high risk for a severe neurodevelopmental disorder with or without endocrine symptoms. Here, we report a 4-week-old new-born with the novel *de novo* missense variant F747S with a so far not described prominent jittering phenotype in addition to symptoms previously reported for *CACNA1D* mutations including developmental delay, elevated aldosterone level and transient hypoglycemia. We confirmed the pathogenicity of this variant in whole-cell patch-clamp experiments with wild-type and F747S mutant channels heterologously expressed together with α2δ1 and cytosolic β3 or membrane-bound β2a subunits. Mutation F747S caused the quantitatively largest shift in the voltage dependence of activation (−28 mV) reported so far for *CACNA1D* germline mutations. It also shifted inactivation to more negative voltages, slowed the time course of current inactivation and slowed current deactivation upon repolarization with both co-expressed β-subunits. In silico modelling and molecular docking, simulations revealed that this gain-of-function phenotype can be explained by formation of a novel inter-domain hydrogen bond between mutant residues S747 (IIS6) with N1145 (IIIS6) stabilizing selectively the activated open channel state. F747S displayed 2–6-fold increased sensitivity for the L-type Ca^2+^ channel blocker isradipine compared to wild type. Our data confirm the pathogenicity of the F747S variant with very strong gain-of-function gating changes, which may contribute to the novel jittering phenotype. Increased sensitivity for isradipine suggests this drug for potential symptomatic off-label treatment for carriers of this mutation.

## Introduction

Voltage-gated Ca^2+^-channel (Cav) are expressed in all electrically excitable cells and play a key role in most physiological processes (1). This is enabled by substantial structural and functional diversity due to the existence of ten different genes encoding their pore-forming α1-subunits and further fine tuning of channel activity and subcellular targeting by associated auxiliary subunits, interacting proteins and posttranslational modifications (1–4). Due to their elementary role for controlling voltage-gated Ca^2+^ entry and cellular excitability, even minor changes in channel function can cause human disease (5). In addition to inherited genetic defects, next-generation sequencing (NGS) techniques have more recently also enabled the detection of sporadic Ca^2+^ channelopathies, which have provided unique insights into the disease mechanisms triggered by Cav dysfunction in humans. Ca^2+^ channelopathies have meanwhile been reported for all 10 α1-subunit isoforms (5,6). Together with the emerging high-resolution cryo-electron microscopy (EM) structures of their pore-forming α1-subunits (7–11), human disease mutations associated with altered channel gating also provide valuable insight into the molecular mechanisms of channel function on the atomic level (12–15).

NGS-based genetic diagnostics continuously enable the discovery of high-risk mutations for rare but also more common conditions. Therefore, an increasing proportion of cases with certain neuropsychiatric and neurodevelopmental disorders, childhood epilepsies, autism spectrum disorders (ASD) and intellectual disability can be attributed to large-effect pathogenic variants, such as about 15% in ASD and >30% in individuals with severe, undiagnosed developmental disorders or epilepsy patients referred to diagnostic NGS (5). Identification of rare, large-effect causal pathogenic variants (16–20) may not only help to confirm the genetic cause of a disease but can also provide hints about potential treatments, including repurposing of existing drugs (21).

We (22,23) and others (24) have recently provided increasing evidence that protein-coding *de novo* germline mutations in the *CACNA1D* gene of the pore-forming Cav1.3 α1-subunit cause high risk or are even causal for a neurodevelopmental phenotype. Initial reports described such *CACNA1D* mutations in two individuals with ASD (A749G, G407R) (14,25,26), followed by reports of two other mutations (G403D, I750M) (24,27) in three severely affected newborns with a predominantly endocrine phenotype of primary aldosteronism and/or hyperinsulinemic hypoglycemia and additional neurological abnormalities (PASNA, OMIM #615474). In patch-clamp studies with these human α1-subunit mutations in heterologous expression systems, characteristic gating changes enabling a gain of channel function were found for all of them. These typical gating changes recently allowed us to classify another mutation, S652L, as pathogenic in homozygotic twins affected by a severe developmental disorder without endocrine symptoms, which was not considered pathogenic in the original study (15). The publications from these studies raised substantial awareness in clinicians for the potential role of *CACNA1D* missense variants as a cause for a neurodevelopmental disorder with or without endocrine symptoms and allowed to identify other mutations (L271H, A749T) in three additional individuals within this disease spectrum (22,28,29). However, it is currently unclear if in addition to the most frequently observed symptoms (developmental delay, hypotonia, ASD, autoaggressive behaviors, intellectual impairment, hypoglycemia, hypertension/hyperaldosteronism), which can vary with respect to their manifestation and intensity in an affected individual (22), other prominent features could guide clinical diagnosis.

Here, we describe a male newborn with unusual severe jittering as the dominant symptom, in addition to symptoms already described in other patients. *CACNA1D* variant F747S was identified as the most likely genetic cause. We therefore examined if this variant also induces gating changes that allow its classification as pathogenic. We also investigated if qualitative or quantitative differences in gating compared to previously described mutations could explain the particularly severe clinical course, which required mechanical ventilation and nasogastric feeding. Moreover, we evaluated if gating changes typical for pathogenic variants are affected by β-subunits stabilizing different gating kinetics by themselves. In addition, we also determined if the mutation-induced alterations in channel gating affect the sensitivity for dihydropyridine (DHP) Ca^2+^ channel blockers, which are obvious candidates for symptomatic treatment of affected individuals with *CACNA1D* gain-of-function mutations.

## Results

### Clinical report

The patient was a 4-week-old male infant admitted to a neonatal intensive care unit after birth with very unusual jitteriness triggered upon contact. He was born after an uneventful pregnancy. The unusual jittering was induced upon routine handling of the baby, which complicated care by nurses. Seizures and drug abuse by the mother during pregnancy could be ruled out as a cause of these symptoms. The child was unable to suck and swallow and to breathe efficiently, which required continuous positive airway pressure (CPAP) treatment and nasogastric feeding. At 7 weeks of age the baby was weaned off CPAP but still required oxygen, nasogastric feeding and was still hypertensive. Jitteriness remained unaltered. After initial episodes of hypoglycemia, pulmonary hypertension and arterial hypertension were also diagnosed with high blood pressure values up to 118/80 mmHg. Hyperaldosteronism with elevated plasma aldosterone (109 ng/dl, reference 2–70 ng/dl) and low renin (0.10 ng/ml/h, reference 0.25–5.82 ng/ml/h) was also documented. The combination of a severe neurological phenotype in combination with congenital hypoglycemia and hyperaldosteronism has been described before in four children with primary aldosteronism with neurological abnormalities (PASNA, OMIM #615474) (24). However, the main clinical symptom found in this case, the pronounced jitteriness, has not been described before. PASNA is part of a wider disease spectrum caused by *de novo* gain-of-function missense variants in the *CACNA1D* gene encoding the pore-forming α1-subunit of Cav1.3 L-type Ca^2+^ channels with 9 such *CACNA1D* mutations so far characterized in 12 individuals (22). Therefore, genetic testing of potential risk genes was performed, revealing the *de novo CACNA1D* variant c.2300T>C (NC_000023.11: g.53764487T>C (hg38, GRCh38)) predicted to cause a p.Phe767Ser mutation (reference sequence: NM_000720.3). It is absent in 125 748 exomes including 60 146 exomes from unrelated control individuals in the gnomAD database (https://gnomad.broadinstitute.org/). This variant corresponds to variant p.PheF747Ser (F747S) in the reference sequence EU363339 of the pore-forming Cav1.3 α1-subunit construct used in this and our previous work (22). It has been submitted to the Leiden Open Variation Database (LOVD 3.0) as genomic variant #0000870940 (https://databases.lovd.nl/shared/variants/0000870940).

Due to this novel phenotype and the severity of the case, we performed a detailed functional analysis of the F747S variant using whole-cell patch-clamp recordings in tsA-201 cells. Demonstration of gating changes permitting channel gain-of-function as previously shown for other high risk *CACNA1D* variants can provide proof for its pathogenicity and may reveal functional changes accounting for the novel and severe phenotype.

### F747S causes pronounced changes in the voltage-dependence of channel gating, channel inactivation and deactivation kinetics

Cav1.3 channel gating is tightly controlled by both, alternative splicing (in particular in the C-terminal tail) (30,31) and the β-subunit isoform forming part of the channel complex (1,32). C-terminal splicing generates a short variant with stronger voltage-sensitivity, increased single channel open probability and more pronounced Ca^2+^-dependent inactivation (30,31). β-subunits also affect gating of L-type channels. In contrast to cytosolic β subunits (β1, β2b, β2c, β2d, β3, β4), the membrane anchored β2a and β2e subunits stabilize slow inactivation behavior of L-type channels (32–35) and may therefore also affect gating changes induced by pathogenic variants. Since we have previously shown that pathogenic *CACNA1D* gain-of-function gating changes are similar in both C-terminally long and short Cav1.3 α1- subunit splice variants, we now analyzed the effects of F747S in C-terminally long Cav1.3 channels (Cav1.3_L_) (31) not only in association with cytosolic β3- but also with membrane-bound β2a-subunits.

**Figure 1:**
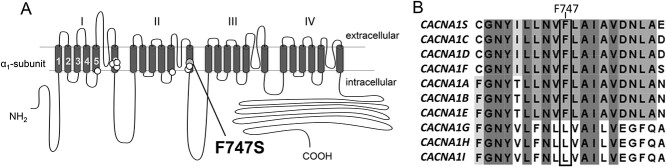
Transmembrane topology of the pore-forming α1-subunit of the Cav1.3 channel. (**A**) In each of the four homologous repeats (I–IV) the segments 1–4 comprise the voltage-sensing domain and segments 5–6 together with their connecting loop form the ion conducting pore (see also Fig. 6 below for a homology model of the channel). All four cytoplasmic ends of S6 together form the activation gate. The position of *de novo* variant F747S (grey circle) is indicated together with other pathogenic germline missense mutations functionally characterized so far (open circles, see main text for details). (**B**) Sequence alignment of parts of the IIS6 segment of all human α1-subunits (gene names are given). The box indicates the position of F747. Amino acid conservation in all (dark grey) or some (light grey) α1-subunits is indicated.

F747 is located within the cytoplasmic portion of the activation gate formed by the IIS6 helix (Fig. 1A) and is conserved among all α1-subunits of the Cav1 and Cav2 family (Fig. 1B). It is in close proximity to other high-risk *CACNA1D* variants found in two individuals with a neurodevelopmental phenotype (A749T) (22,29), a child with ASD (A749G) (14) and a PASNA patient (I750M) (24).

**Figure 2:**
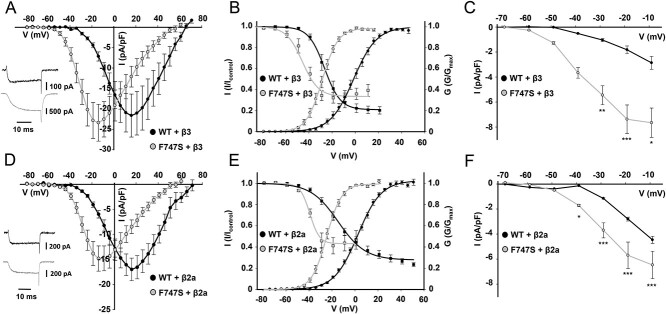
Voltage-dependence of WT and F747S channel gating. WT or mutant channels were coexpressed with β3 (**A–C**) or β2a (**D–F**) together with α2δ1-subunits. Current–voltage relationships (A, D) were generated with 25-ms depolarizing square test pulses from a holding potential of −89 mV in 5 mV increments to the indicated test potentials. Current densities are shown (current normalized to the cell size, pA/pF). Representative traces upon depolarization to *V*_max_ are shown in the insets. (B, E) Normalized steady-state activation (circles; obtained from the I**–**V-relationships) and inactivation curves (squares) were obtained as described in methods. For detailed parameters, n-numbers and statistics see Table 1. (C, F) Window currents calculated from the current densities in I-V-curves and steady-state inactivation parameters as described in methods. Statistics: one-way ANOVA with Bonferroni post hoc test. Data are presented as mean ± SEM from at least 3–5 independent transfections.

In western blots, total F747S α1-subunit protein expressed together with β3 and α2δ1 in tsA-201 cells migrated with the expected molecular mass indistinguishable from WT but with an about 13-fold higher total protein expression level (*n* = 3, Supplementary Material, Fig. S2). However, this did not result in an alteration of maximal current density (Fig. 2A and D). As previously observed for the pathogenic Cav1.3-mutations (36), we could not observe ON-gating currents as in WT (see insets in Fig. 2A and D) as a parameter for quantification for channel surface expression. Therefore, no attempts were made to quantify changes in plasmalemmal targeting of F747S. However, F747S induced pronounced and highly significant shifts of the voltage dependence of activation (by ~28 mV) and steady-state inactivation (by ~17–23 mV) to more negative voltages, independent of the co-expressed β-subunit isoform (Fig. 2B and E; Table 1). The increase in voltage-sensitivity was also evident from the significantly smaller slope factors (*k*) obtained from fitting the activation and inactivation data to a Boltzmann equation (Table 1). This shift in voltage-dependent gating resulted in a significant increase in non-inactivating window current, which predicts a constant background Ca^2+^-influx in excitable cells at negative potentials as low as −40 mV (Fig. 2C and F). Note that this voltage is equivalent to an about 18 mV more hyperpolarized potential with physiological extracellular Ca^2+^ (2 mm) as charge carrier, since 15 mm extracellular Ca^2+^ in our recording solution shifts the voltage dependence to more positive voltages (31).

**Table 1:** Steady-state activation and inactivation parameters of Cav1.3 WT and F747S channels

α1-subunit	Activation				Inactivation			
	*V*_0.5_ (mV)	*k* (mV)	*V*_rev_ (mV)	*n*	*V*_0.5, inact_ (mV)	*k* (mV)	Non-inactivating (%)	*n*
WT + β3	−0.12 ± 1.32	9.14 ± 0.18	62.82 ± 1.99	13	−26.02 ± 2.31	6.07 ± 0.35	20.48 ± 3.45	6
F747S + β3	−28.5 ± 1.48^*^^*^^*^	5.97 ± 0.15^*^^*^^*^	41.64 ± 1.71^*^^*^^*^	13	−42.88 ± 1.80^*^^*^^*^	4.93 ± 0.18^*^	31.24 ± 4.86	9
WT + β2a	1.98 ± 0.97	10.08 ± 0.14	66.41 ± 2.85	13	−16.22 ± 2.55	10.12 ± 0.48	30.30 ± 4.22	9
F747S + β2a	−24.68 ± 1.41^*^^*^^*^	5.94 ± 0.16^*^^*^^*^	44.79 ± 1.84^*^^*^^*^	10	−38.94 ± 1.00^*^^*^^*^	4.04 ± 0.16^*^^*^^*^	42.48 ± 7.53	8

Parameters were obtained from fitting normalized current–voltage activation (*I*/*I*_max_) and steady-state inactivation curves (*I*/*I*_control_) to Boltzmann relationship, as described in methods. All values are presented as mean ± SEM from at least 3–5 independent transfections and number of recordings (*n*) are indicated. Statistics: unpaired Student’s *t*-test (Bonferroni-corrected for multiple testing of two different β-subunits, ^*^P < 0.05, ^*^^*^^*^P < 0.001 compared to the wild-type (WT). *V*_0.5_, voltage of half maximal activation; *V*_0.5,inact_: voltage of half maximal inactivation; *k*, slope factor; *V*_rev_, extrapolated reversal potential.

**Figure 3:**
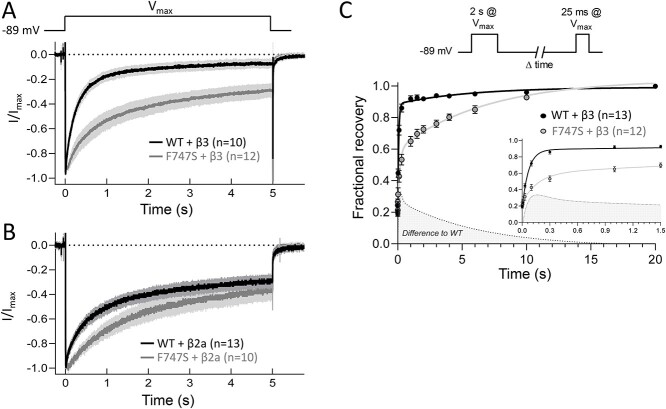
Inactivation time course of Cav1.3 WT and F747S channels. Inactivation kinetics of normalized Cav1.3 WT (black traces) and F747S (grey traces) channel currents during 5-s depolarizing pulses to *V*_max_ from a HP of −89 mV. Channels were co-expressed with β3- (**A**) or β2a- (**B**) together with α2δ1-subunits. Curves represent the means ± SEM from at least 3–5 independent transfections. For statistical significance of inactivation after pre-specified time points see Table 2. Note the slowing of the inactivation time course induced by β2a-subunits (B). Parameters obtained by fitting individual inactivation curves to exponential decay are given in Table 3. (**C**) Time courses of recovery from inactivation of Cav1.3-WT (black) and F747S (grey) co-expressed with β3 examined using a two-pulse protocol (top). The current amplitude during the 25 ms control pulse was normalized to peak current evoked by the 2 s prepulse and plotted as a function of recovery time. Data are present as mean ± SEM, n numbers are indicated in parenthesis. Data were fit to a double exponential function (solid lines). The following parameters (means ± SEM) were obtained: WT: *A*_fast_ = 0.72 ± 0.02, *τ*_fast_ = 67.6 ± 5.72 ms, *A*_slow_ = 0.10 ± 0.02, *τ*_slow_ = 5625 ± 3562 ms; F747S: *A*_fast_ = 0.36 ± 0.04, *τ*_fast_ = 165.94 ± 43.7 ms, *A*_slow_ = 0.44 ± 0.04, *τ*_slow_ = 6778.23 ± 1930 ms, extra sum-of-square *F* test, *F* = 72.97, *P* < 0.0001). *A*_fast_, *A*_slow_: fractional amplitude of fast and slow recovering component; *τ*_fast_, *τ*_slow_: time constants of fast and slow recovering component.

**Figure 4:**
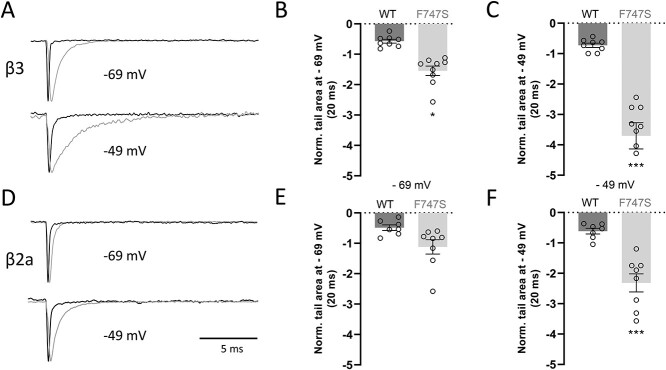
Deactivation kinetics and tail currents of Cav1.3 WT and F747S channels. (**A**, **D**). Representative traces of tail currents recorded in Cav1.3 WT (black) or F747S channels (grey) co-expressed with β3- (A) or β2a- (D) together with α2δ1-subunits during 20-ms repolarizations to −69 mV or − 49 mV after 10-ms test pulses to the reversal potential. To quantify the mutation-induced relative change in Ca^2+^ charge during deactivation, normalized tail currents at both test voltages were integrated over 20 ms and are shown for both −69 mV (**B**, **E**) and −49 mV (**C**, **F**) for WT (dark grey bars) and F747S (light grey bars) co-expressed with β3- (B, C) or β2a -subunits (E, F). Statistics: one-way ANOVA with Bonferroni post-hoc test. Data are presented as mean ± SEM (>3 independent transfections).

Co-expression of WT channels with β3-subunits resulted in a fast inactivation time course during 5 s depolarizations to *V*_max_, which was significantly slower for F747S (Fig. 3A and Tables 2 and 3). As expected, the bi-exponential inactivation of WT channels was also significantly slowed by co-expressed β2a subunits (Fig. 3B and Tables 2 and 3). Unlike WT and independent of the co-expressed β-subunit, inactivation of F747S always followed a monoexponential time course with a time constant (τ) similar to the slow component in WT (Table 3), suggesting that the mutation primarily prevents a fast inactivating phase of inactivation. The gain-of-function phenotype was further supported by the prolongation of channel deactivation during repolarizing voltage-steps after a test pulse to the reversal potential. F747S induced a pronounced slowing of deactivation kinetics in the presence of both β-subunits upon repolarization to either −69 or −49 mV with a 4–5-fold increase in the normalized area of the tail current at −49 mV (Fig. 4). Interestingly, F747S also caused a small but highly reproducible delay of the tail current peak before it decreased with a slower deactivation time course (visible in representative traces in Fig. 4A and D).

**Table 2:** Inactivation time course of Cav1.3 WT and F747S channels

α1-subunit	*r*_50_	*r*_100_	*r*_250_	*r*_500_	*r*_1000_	*r*_5000_	*n*
WT + β3	79.88 ± 3.73	67.83 ± 3.52	47.18 ± 3.70	29.29 ± 3.16	17.94 ± 2.99	8.27 ± 1.89	10
F747S + β3	95.43 ± 1.70^*^^*^	90.01 ± 3.63^*^^*^^*^	79.49 ± 6.25^*^^*^	68.02 ± 7.20^*^^*^^*^	55.45 ± 7.15^*^^*^^*^	29.27 ± 4.76^*^^*^	12
WT + β2a	88.83 ± 1.52	83.56 ± 2.34	69.98 ± 2.51	61.32 ± 3.47	48.27 ± 3.33	26.21 ± 3.50	13
F747S + β2a	97.22 ± 1.59^*^^*^	95.95 ± 1.48^*^^*^^*^	88.20 ± 1.95^*^^*^^*^	80.80 ± 2.98^*^^*^^*^	67.85 ± 4.31^*^^*^	34.13 ± 4.93	10

*r* values represent the remaining *I*_Ca_ (% of the peak inward current) after prespecified time points (50, 100, 250, 500, 1000 and 5000 ms) during a 5 s depolarization pulse to *V*_max_ from the holding potential of −89 mV. All values are presented as means ± SEM from the indicated numbers (*n*) of recordings obtained in at least 3–5 independent transfections. Statistics: unpaired Student’s *t* test (Bonferroni-corrected for multiple testing of two different β-subunits, ^*^P < 0.05, ^*^^*^P < 0.01 ^*^^*^^*^P < 0.001 compared to the WT.

**Table 3:** Exponential inactivation parameters of Cav1.3 WT and F747S channels

α1-subunit	*A*_slow_ (%)	*τ*_slow_ (ms)	*A*_fast_ (%)	*τ*_fast_ (ms)	Non-inactivating (%)	*n*
WT + β3	25.13 ± 4.86	1987.59 ± 465.82	63.74 ± 6.36	227.20 ± 36.52	7.12 ± 2.45	7
F747S + β3	67.48 ± 3.26	1424.08 ± 268.56	–	–	28.27 ± 3.61	12
WT + β2a	41.27 ± 3.72^*^	2070.87 ± 286.85	28.97 ± 2.90^*^^*^^*^	283.48 ± 35.60	24.74 ± 3.33^*^^*^	12
F747S + β2a	65.75 ± 4.94	2176.09 ± 497.13	–	–	31.61 ± 4.59	10

Normalized inactivation currents were individually fitted to an exponential decay. Wild-type (WT) kinetics were best fit with a biexponential decay (+β3: 10 out of 2; +β2a: 12 out of 12: *τ*_fast_, *τ*_slow_: time constants of slow and fast component; *A*_fast_, *A*_slow_: percent of slow and fast inactivating components), whereas inactivation of F747S mutant channels always followed a monoexponential time course. Statistics: WT/β3 versus WT/β2a: unpaired Student’s *t*-test with Bonferroni adjustment of *P*-values for multiple comparisons; ^*^^*^P < 0.01, ^*^^*^^*^P < 0.001; no significant differences were observed between inactivation parameters of F747S channels expressed with β2a- and β3-subunits. All data are presented as mean ± SEM (>3 independent transfections, n indicated the number of recordings).

In addition, F747S also significantly slowed recovery from inactivation induced by a 2-s prepulse to *V*_max_. Slower recovery was primarily caused by a reduction of the contribution of the fast and an increase in the slowly recovering component (for recovery parameters see legend to Fig. 3C). These data clearly show that F747S can induce gating characteristics that permit channel gain-of-function, in particular by a pronounced reduction of the activation threshold when depolarized from negative membrane potentials, enhanced steady-state Ca^2+^ inward current at subthreshold voltages, reduced inactivation during longer depolarizations and prolonged Ca^2+^-influx during repolarization.

The particularly severe and so far unique clinical phenotype could be due to qualitative and/or quantitative changes of Cav1.3 channel function by the mutation. The gating changes induced by F747S do not differ qualitatively from other mutations, because the negative shifts of activation and inactivation gating as well as the additional slowing of inactivation and prolonged deactivation have been described before by us and others (15,37). However, they differ quantitatively as F747S caused the strongest effects on channel function observed so far when compared with other germline mutations. This is illustrated in Table 4 in which we compare gating parameters of germline mutations that were analyzed by us under identical experimental conditions, which allows a direct comparison.

**Table 4:** Effects of different *CACNA1D* missense mutations on Cav1.3 voltage-dependent gating parameters and inactivation kinetics

Mutation	*V*_0.5,act_ (mV)	*V*_0.5, inact_ (mV)	r500%	r5000%	Reference
	Difference (WT, Mutant)				
F747S	**−28.4** (−0.12, −28.5)	−16.9 (−26.02, −42.9)	**−38.7** (29.3, 68.0)	**−21.0** (8.3, 29.3)	This publication
V401L	−16.6 (0.77, −15.8)	−3.9 (−26.1, −30.0)	−9.2 (37.5, 46.7)	−1.6 (15.9, 17.5)	13
S652L	−16.1 (−0.18, −16.3)	**−17.6** (−25.7, −43.3)	14.1 (24.9, 10.8)	3.27 (8.60, 5.33)	15
A749G	−9.75 (−2.55, −12.3)	−15.4 (−25.7, −41.1)	15.6 (22.8, 7.22)	3.0 (6.12, 3.12)	14, 36
I750M	−15.2 (−4.9, −20.1)	−12.4 (−25,7, −38.1)	−15 (28.5, 43.5)	−11.3 (11.0, 22.3)	61
*F747L*	*−17 (1.37, −15.6)*	*−2.3 (−27, −29.3)*	*−34 (29.4, 63.4)*	*−22.4 (9.83, 32.2)*	*36*

Gating parameters of the mutant channels were measured under identical experimental conditions in separate publications (co-expression with β3-subunits) and thus can be directly compared (data from (38) were corrected for a liquid junction potential of −9.3 mV for this table as in the other publications). Data from mutation G407R also analyzed in (14) are not included (missing steady-state inactivation parameters). Values represent the difference of the respective parameter between WT (first number in parenthesis) and the given mutant channel (second number in parenthesis). A negative value indicates a shift of the voltage dependence of activation (*V*_0.5,act_) or inactivation (*V*_0.5,inact_) towards more negative potentials or a slowing of the inactivation time course. Bold numbers indicate parameters with the strongest effect among all germline mutations shown. Mutation F747L is indicated in italics because it has not been reported as a germline mutation but was found as a somatic *de novo* variant in aldosterone-producing adenomas (38). It is shown for comparison to illustrate the weaker gating effects as compared to F747S. *V*_0.5,act_, voltage of half-maximal activation; *V*_0.5,inact_, voltage of half-maximal inactivation; r500 or r5000, remaining current in % of the peak inward current after 500 or 5000 ms during a 5 s long depolarization to the voltage of maximal activation (*V*_max_).

### Effects of the F747S mutation and β-subunit composition on dihydropyridine drug sensitivity

**Figure 5:**
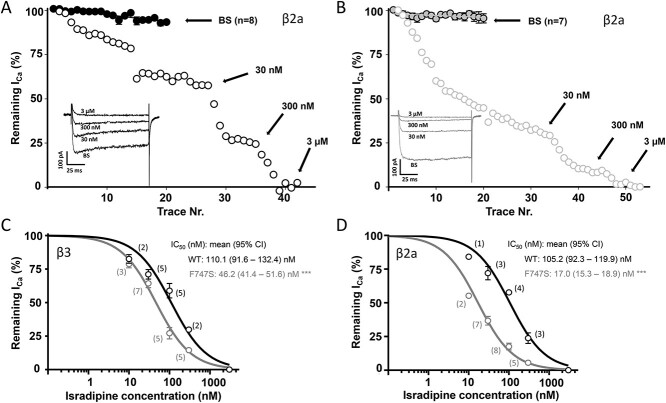
Inhibition of β3- and β2a-containing Cav1.3 WT and F747S channels by isradipine. (**A**, **B**) Representative experiment for inhibition of peak inward *I*_Ca_ (with co-expressed β2a) through Cav1.3 WT (A, black open circles) or F747S channels (B, grey open circles) by 30 and 300 nm isradipine (arrows indicate steady-state inhibition) during continuous 100-ms depolarizations (0.1 Hz) from the HP to *V*_max_. Insets: Representative current traces obtained before (bath solution, BS) and after maximal inhibition by the indicated isradipine concentrations (3 μm were used for full block at the end of each experiment). The mean time course of current decay in the absence of drug (linear current run-down; BS only) is also shown as the mean (±SEM) from all control experiments measured in parallel with the WT and mutant channels on different days (A, B, filled circles). Run-down-corrected concentration-inhibition curves are shown for both β3- (**C**) and β2a-containing channel complexes (**D**) for WT (black) and F747S channels (grey). Data are presented as means ± SEM. Curves were computer-fitted as described in Methods using a Hill slope of 1 with top and bottom fixed (bottom = 0; top = 100). Concentrations for half maximal inhibition (IC_50_) are given as means with the 95% confidence interval (CI). The number of experiments for each concentration is indicated. Statistical significance (F747S versus WT) was calculated using the extra sum-of-square *F*-test; ^*^^*^^*^P < 0.001. Run-down for β3-containing channel complexes was faster (slope; WT: −1.5383, F747S: −0.4941) compared to β2a (WT: −0.4046, F747S: −0.1006).

Because F747S can induce a gain of channel function, pharmacological inhibition of excess Ca^2+^ influx may improve symptoms, in particular jittering, in this child. Brain-permeable organic Ca^2+^ channel blockers, particularly dihydropyridines (DHPs) represent such a therapeutic option, because they are widely used as well-tolerated antihypertensive drugs and are readily available for off-label use. However, these drugs act in a highly voltage-dependent manner (39,40). Therefore, the pronounced gating changes may affect the apparent sensitivity towards DHPs, which may also have implications on its therapeutic usefulness. We have recently shown that mutations S652L and V401L, when coexpressed with β3-subunits, can increase the apparent sensitivity for the DHP isradipine (13,15) whereas other mutations (G407R, unpublished data) can decrease it. Moreover, it is unknown if the slowing of inactivation caused by β2a-subunits can affect isradipine sensitivity of WT and mutant channels. To address these questions, we perfused tsA-201 cells transfected with WT and F747S channels co-expressed with either β3- or β2a-subunits with extracellular solution (control to quantify current run-down) or increasing concentrations of isradipine under identical experimental conditions (holding potential −89 mV, 100 ms test pulses, 0.1 Hz) to determine run-down corrected current inhibition (Fig. 5A and B, illustrated for β2a co-expression). F747S mutant channels were significantly more sensitive to isradipine than WT with both β-subunits (*P* < 0.001, extra sum-of-square *F*-test), an effect more pronounced with co-expressed β2a-subunits (~6-fold) than with β3 (~2.4-fold) (Fig. 5C and D). DHPs preferentially act on inactivated channel states, which could explain the higher sensitivity of mutant channels due to their more negative inactivation voltage range. While a more detailed pharmacological analysis is required to completely elucidate the mechanism for this sensitivity increase, the potential clinical implications of this finding are of interest. DHPs are expected to counteract the enhanced signaling of mutant Cav1.3 channels and may therefore improve symptoms (such as jitteriness) in affected patients.

### In silico modelling and molecular dynamics simulations of the F747S mutation in different gating states of the Cav1.3 α1-subunit

**Figure 6:**
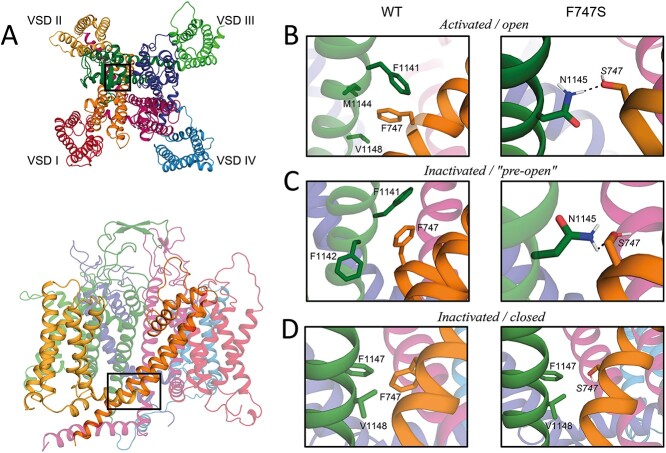
Molecular modeling of the F747S mutation in homology models of different conformational states of the Cav1.3 α1-subunit. (**A**) Top (top panel) or side view (bottom panel) of the activated-open channel conformation of the Cav1.3 α1-subunit. The box highlights the region of interaction between the newly formed hydrogen bond between F747S (IIS6, orange) and N1145 (IIIS6, green). VSD, voltage-sensing domains. (**B**) Detailed view of the F747S—N1154 interaction in the predicted activated-open channel conformation of mutant channels. (**C**) This novel hydrogen bond also forms in a recently published Cav1.3 α1-subunit structure (11) in which the activation gate assumes a conformation with structural features of both the open and inactivated-closed conformation and may represent a "pre-open" channel state (Supplementary Material, Fig. S1). (**D**) The F747S—N1154 is absent in the inactivated (and resting, not illustrated) channel conformation. For further details for modeling of the different states, see section Methods.

In order to explain the observed biophysical property changes resulting from the mutation, we modelled and simulated the structures of the Cav1.3 α1-subunit of the WT and the F747S mutant (Fig. 6A–D). Unlike with previous mutations, we not only analyzed the effects of the mutation in the inactivated-closed state (voltage-sensors "up," activation gate closed; state assumed for high-resolution structure of channels purified at zero membrane potentials), but also in the activated open (voltage-sensors "up," activation gate open) and resting state (voltage-sensors "down," activation gate closed). This was possible by generating homology models based on the available high-resolution cryo-electron microscopy (EM) structures of voltage-gated Ca^2+^- and Na^+^-channel pore-forming subunits captured in different states of the gating cycle (activated open: open state-stabilized Nav1.5 α-subunit; inactivated closed: Cav1.1 α1-subunit structure; resting state: NavAb disulfide crosslinked mutant α-subunit; see section Methods for details). While residue F747 is mainly surrounded by hydrophobic and aromatic residues of the neighboring S5 and S6 helices, the introduction of a polar serine residue at this position allows the formation of hydrogen bond interactions. Indeed, we find an additional inter-domain hydrogen interaction between mutant residue S747 (IIS6) and N1145 (IIIS6), which can form in the activated-open channel conformation (Fig. 6B) but not in the inactivated closed (Fig. 6D) or resting state (not shown). Therefore, this state-dependent interaction of the mutant channel is expected to stabilize the open channel state, which can explain the prolongation of tail currents, the slowing of the inactivation time course and the negative shift in the voltage-dependence of channel activation. Very recently, the cryo-EM-structure of the Cav1.3 Ca^2+^ channel complex has been published (11). We therefore compared this structure with our modeled structures. Surprisingly, the activation gate of the Cav1.3 α1-subunit assumes a conformation with structural features of both the open and the inactivated channel state. In the cryo-EM structure of related Cav1.1 α1-subunits (41) and in our Cav1.3 homology model of the inactivated closed state derived from it, four phenylalanine residues point inwards and thereby tightly seal the orifice of the activation gate for permeating Ca^2+^ ions (Supplementary Material, Fig. S1A). In contrast, in the cryo-EM structure of Cav1.3, these phenylalanines have moved outside, pointing away from the ion-conducting pathway (Supplementary Material, Fig. S1B, upper panel). Therefore, the seal is lost and water molecules can occupy the gate (Supplementary Material, Fig. S1B, bottom, cyan surface). This arrangement of phenylalanines closely resembles their conformation in the predicted activated open state structure (Supplementary Material, Fig. S1C). However, the activation gate still appears to be closed as the cytoplasmic portions of the S6-helices have not widened the gate as seen in for the activated open conformation (Supplementary Material, Fig. S1C). We therefore propose that this cryo-EM structure represents an inactivated channel state as expected for WT Cav1.3 channels purified and analyzed at zero membrane potential. However, the activation gate assumes a different conformation than in the inactivated closed state of Cav1.1 α1 and the corresponding inactivated closed state Cav1.3 homology model. Instead, the gate likely assumes a "wetted" conformation that may more easily transition into the open state (therefore termed "pre-open" in Fig. 6C and Supplementary Material, Fig. S1B). Like in the activated open activation gate (Fig. 6B), the hydrogen bond between the mutant S747 and N1145 can also form (Fig. 6C), suggesting that this (likely pre-open) state of the activation gate is also stabilized by the mutation.

## Discussion

Here, we report the *CACNA1D* variant F747S as a novel pathogenic mutation in a newborn with prominent jittering. This prominent phenotype was induced and aggravated upon interaction with the child and thus complicated routine care. This unusual symptom expands the phenotypic spectrum of symptoms reported so far in 12 individuals affected by a total of 9 *CACNA1D* mutations (22). We and others have previously functionally analyzed 6 of them, consistently revealing very similar and characteristic gating changes, with all enabling a channel gain-of-function. Here, we show that these gating changes are qualitatively the same in the F747S variant. The main characteristic is the channel activation at more negative potentials, further shifting the already negative voltage operation range of wild-type Cav1.3 channels (39,42) towards more hyperpolarized potentials. This also predicts a constant background Ca^2+^ influx (window current) at subthreshold potentials, i.e. even when neurons are not actively firing action potentials. Furthermore, we have previously shown that the slowing of channel deactivation significantly increased Ca^2+^ load during the repolarizations of simulated action potential firing (15). Although qualitatively similar, the magnitude of the gating defects, i.e. shift of the voltage dependent activation and inactivation gating to more negative voltages and slowing of channel inactivation kinetics, is larger for F747S than observed for the other mutations analyzed by us previously under identical experimental conditions. This is especially true for the voltage-dependence of activation (Table 4) favoring channel opening at about 12 mV more negative voltages than in other germline mutants. In this comparison, we did not include the inactivation-deficient variant G407R, for which voltage-dependence of inactivation could not be determined (14) and activation voltage for Cav1.3 is unchanged (14).

However, we also observed another striking gating phenotype not previously reported for a *CACNA1D* variant. F747S strongly reduced the recovery from channel inactivation, which could favor accumulation of channels in inactivated states during prolonged neuronal firing. This illustrates that, in addition to permitting channel gain of function, these disease-associated germline *CACNA1D* mutations may also cause a reduction of channel availability during certain firing patterns by shifting steady-state inactivation to more negative voltages or by slowing recovery from inactivation. Notably, we have previously shown that mutation V401L, described in a severely affected patient but without the jittering phenotype, also induces negative shifts in the channel's voltage-dependence and slows inactivation, but, in contrast to F747S, promotes recovery from inactivation (13). Although a detailed phenotype–genotype relationship still requires a larger number of cases, our findings strongly suggest that the unusually high voltage-sensitivity for channel opening, in combination with slower inactivation and deactivation gating, and/or a pronounced slowing of recovery from inactivation could favor the more severe clinical phenotype observed in this newborn. To verify this assumption and to understand how these gating changes can affect motor function requires generation of suitable mouse knock-in models allowing analysis of mutant channels in their native environment and of altered brain function *in vivo*. For mutation A749G (14) such work is in progress in our laboratory.

Clinicians should consider *CACNA1D* as high-risk gene for newborns with a prominent jittering phenotype and immediately screen the child for additional symptoms associated with *CACNA1D* mutations, in particular those of acute relevance such as high risk for hyperinsulinemic hypoglycemia and hypertension due to primary aldosteronism (22).

Somatic *CACNA1D* variants are found in about 10–30% of APAs (43,44) and up to about 60% in their precursor lesions, aldosterone-producing cell clusters (APCCs) (45). Such mutations are diagnosed much more frequently than germline mutations. We have recently collected information on ~60 published somatic *CACNA1D* mutations reported in APAs/APCCs to generate a map that could help with the interpretation of the pathogenic potential of a new germline variant (22). This is based on the observation that 5 of the 9 germline mutations were also found in somatic APAs/APCCs (S652L, V401L, I750M) or in identical positions of an APAs/APCCs mutation (G403D, V259A) (22). This is also true for F747S, because substitutions of F747 to cysteine, leucine, valine and serine have recurrently been reported in APAs (22,43). However, we have also shown in a previous study that different amino acid substitutions in the same position can have opposite effects on gating and therefore also different pathogenic potential. S652L, identified in homozygotic twins with a severe neurodevelopmental syndrome, induces a typical gain of channel function while S652W, which is also reported in healthy controls, induces (nonpathogenic) gating changes compatible with a minor loss of channel function (15) (S672 in gnomAD database reference sequence NM_000720). In the case of F747 variants, F747L (36) causes weaker but qualitatively very similar gain-of-function gating changes compared to F747S, indicating that substitution of the phenylalanine in this position by either a polar serine or a hydrophobic leucine residue helps stabilizing open channels.

Unlike earlier studies, we performed homology modelling in combination with molecular dynamics simulations of F747S not only in a predicted inactivated channel state but also in a predicted resting and an open state. This was enabled by recent publications of voltage-gated ion channel high resolution structures not only in the inactivated-closed state but also with the activation gate stabilized in open and resting conformations of the channel (41,46–48). This now allowed us to model mutation-induced structural changes in different conformations and thus to make more precise predictions about the stabilization or destabilization of particular channel states during the activity cycle of the channel. We show that compared to WT phenylalanine residue F747, the mutant serine S747 can form an additional inter-domain hydrogen bond with residue N1145 located in the IIIS6 helix. This strongly suggests that this interaction stabilizes an open conformation of the activation gate. Notably, this stabilizing inter-domain hydrogen bond interaction occurred in the activated state with activation gate open but was not observed in the inactivated state with the activation gate sealed. The observed stabilization of the activated open state can explain the experimentally observed shift in voltage-dependence as well as the slowing of channel inactivation and deactivation. Our approach therefore emphasizes analysis of the structural consequences of disease variants in molecular models of different channel conformations.

Moreover, our modeling studies also strongly suggest that in a recently published cryo-EM structure of the inactivated Cav1.3 channel complex the conformation of the activation gate clearly differs from the cryo-EM structure of Cav1.1 and our inactivated-closed state Cav1.3 homology model, lacking the tight hydrophobic seal of the gate by inwardly-oriented phenylalanines and allowing water molecules to enter the gate. The arrangement of these phenylalanines is similar to their conformation in the predicted activated open activation gate structure. Although this conformation is captured in a putative inactivated state of the channel, this conformation may also exist after the voltage sensors have moved "up" by depolarization but before channel opening. In this case, it would comprise a pre-open state with a reduced energy barrier for entering the open state, which could explain the more negative activation voltage-range characteristic for Cav1.3 channels (49,50). Formation of the S747—N1145 hydrogen bond in the mutant in the open and the proposed pre-open state could favor open channel states and more efficient coupling of voltage-sensor movements to gate opening.

The gating properties of Cav1.3 channels are affected by alternative splicing both within and outside the long C-terminal tail of the α1-subunit with profound effects on voltage-dependent gating and Ca^2+^-dependent inactivation. We have previously shown that alternative splicing does not prevent effects on the gating changes of several germline disease mutations (13,36,51). However, channel gating is also tightly controlled by the β-subunit isoform associated with the channel. In contrast to cytosolic β-subunits, such as the β3-subunit which is abundantly expressed in the brain (52), the membrane-anchored β2 splice variants β2a and β2e induce a pronounced slowing of Cav1.3 channel inactivation. This may affect both, mutation-induced gating changes as well as the pharmacological sensitivity to state-dependent DHP Ca^2+^ channel blockers, such as isradipine (40). We therefore compared the properties of channels co-expressed with β3-subunits (routinely used in our previous studies) head-to-head with co-expressed β2a-subunits (both always together with α2δ1-subunits to allow analysis of physiological channel complexes) (41). This is of relevance because we have previously shown that these membrane-anchored β-subunit variants can form a substantial fraction of β-subunit transcripts in neurons, such as substantia nigra dopamine neurons (35). We found that the pronounced shifts of voltage-dependent gating induced by the F747S mutation also occurred in β2a-containing channel complexes, suggesting that pathogenic functional changes can occur also with membrane-bound β-subunits stabilizing slow kinetics. β2a slowed the biexponential time course of inactivation by significantly decreasing the contribution of the fast inactivating component (Table 3) without significant changes of *τ*_fast_ and *τ*_slow_. Mutation F747S eliminated fast inactivation leading to a mono-exponential inactivation time course with a time constant similar to the slowly inactivating Cav1.3 current components. This suggests that F747S favors slow channel inactivation limiting a further slowing by β2a.

We also discovered a novel gating change induced by the F747S mutation. It caused a "hook" in the tail currents upon repolarizations to negative voltages after channel activation induced by a short test pulse to the reversal potential. This hook corresponds to a small but highly reproducible delay of the tail current peak before it decreased with a slower deactivation time course. The phenomenon of L-type channel re-openings has previously been explained by the existence of closed states outside the activation pathway from which the channel reopens upon repolarization (53). Mechanistically, positively charged blocking particles (54) or blocking cations, such as Cd^2+^ (55) have been proposed to occlude the pore during depolarization followed by channel reopening when block by these particles is relieved by repolarization of the cell to negative potentials. It is therefore possible that the F747S mutation, once closed after opening, can favor a channel conformation from which channels can reopen upon repolarization. Detailed single channel analysis of F747S in comparison with wild-type channels is required to investigate this possibility.

We also found that mutation F747S induces profound changes in the pharmacological properties of Cav1.3. Mutant channels exhibited higher sensitivity for the DHP Ca^2+^ channel blocker isradipine. This may in part be due to the preferential binding of DHPs to inactivated channel states, which are favored by the more negative inactivation voltage range of F747S channels. However, this increase in potency was even more pronounced with co-expressed β2a although β2a did not induce more negative steady-state inactivation and even inhibited, as expected, inactivation during depolarization. It is therefore possible that β2a-induced conformational changes facilitate binding or drug access to the binding site. Although this needs to be addressed in further studies, the clinical implications of our finding are important because it indicates that the drug sensitivity is maintained, and even increased, for the DHP isradipine, and likely other DHPs. DHPs therefore remain an option for symptomatic treatment of this and other patients affected by DHP-sensitive or even hypersensitive mutations, such as shown here for F747S. However, this therapy remains a challenge especially for newborns and patients with difficult adherence to oral drug therapy. Unfortunately, elimination half-lives of DHPs (except for amlodipine, which has only limited brain exposure) (56) are short and require extended release formulations which are only available for adult dosing. Studies addressing this issue are currently underway.

## Materials and Methods

### Complementary DNA constructs

Human wild-type (WT) Cav1.3 α1-subunit (reference sequence EU363339) comprising alternative exons 8a and 42 (long C-terminal splice variant, Cav1.3_L_) was previously cloned into the pGFPminus vector (no GFP tag, CMV promoter) (31). Mutation F747S was introduced into Cav1.3_L_ using standard polymerase chain reaction approaches and verified by Sanger sequencing (Eurofins Genomics, Ebersberg, Germany). To introduce F747S variant into Cav1.3_L_, splicing by overlap extension (SOE)-PCR was used. Briefly, nt 1673-4047 of Cav1.3_L_ were PCR amplified with overlapping primers (primer pairs 1a and 1b) introducing the point mutation T>C (Phe > Ser) at position nt 2240 in separate PCR reactions (PCR 1A and PCR 1B) using Cav1.3_L_ as template. The two separate PCR products were then used as templates for the final PCR reaction (PCR 2) with primer pair 2. This fragment was then AauI/HindIII digested and cloned into respective sites of Cav1.3_L_ yielding to Cav1.3 F747S. The following primer pairs were used for SOE PCR of F747S construct (purchased from Eurofins, Ebersberg, Germany):

primer pair 1a: AauI (BrsGI) fwd: 5′-CCAACAAAGTCCTCTTGGCTCTGTTC-3′, F747S SOE rev: 5′-CTTTCAGCATCAGCCAAATTGTCTACAGCGATGGCCAAGGAGACATTCAGTAGAATATAG-3′ (607 bp); primer pair 1b: F747S SOE fwd: 5′-CTATATTCTACTGAATGTCTCCTTGGCCATCGCTGTAGACAATTTGGCTGATGCTGAAAG-3′, HindIII rev: 5′-ATAGATGAAGAACAGCATGGCTATGAGG-3′ (1828 bp); primer pair 2 AauI (BrsGI) fwd and HindIII rev (2375 bp).

Auxiliary subunits: rat β2a (M80545), rat β3 (NM_012828) and rabbit α2δ-1 (NM_001082276).

### Cell culture and transfection

TsA-201 cells were obtained from the European Collection of Authenticated Cell Cultures (ECACC, catalogue number 96121229, lot number 13D034) at passage 6. Cell stocks of passage 8 were frozen and cultures were re-expanded from stocks for not more than 20 passages. Cell cultures were tested negative (Universal Mycoplasma Detection Kit 30-1012 K, American Type Culture Collection) for mycoplasma infection. For electrophysiological experiments tsA-201 cells were maintained and cultured in Dulbecco’s modified Eagle’s medium (DMEM; Sigma-Aldrich, D6546) completed with 10% FBS (Gibco, 10270-106), 2 mm l-glutamine (Gibco, 25030-032), penicillin (10 U/ml; Sigma-Aldrich, P3032) and streptomycin (10 μg/ml; Sigma-Aldrich, S6501) at 37°C and 5% CO_2_ in a humidified incubator. Cells were transiently transfected with 3 μg of WT or mutated Cav1.3 α1, 2 μg β2a (rat, M80545) (50) or β3 (rat, NM_012828) (50) and 2.5 μg α2δ-1 subunits (rabbit, NM_001082276) (50) using the Ca^2+^-phosphate precipitation method always including EGFP cDNA (1.5 μg) as a transfection marker. All data were obtained from at least 3 independent transfections.

### Western blots

Western blot immunodetection of α1-subunits in tsA-201 membrane preparations was performed as previously described (15). Primary antibodies: rabbit anti-Cav1.3 (Alomone labs, ACC-005; 1:1000), mouse anti-α-tubulin (Merck Millipore, CP06; 1:25 000 or 1:30 000) and rabbit anti-GFP (Thermo Fisher Scientific, A6455; 1:10 000). Secondary antibodies: goat anti-rabbit (Sigma-Aldrich, A0545; 1:20 000) and goat anti-mouse (Thermo Fisher Scientific, 31430; 1:8000). However, we optimized normalization of α1-subunit protein expression as follows: To account for variability in transfection efficiency with different cDNA constructs, we transiently transfected tsA-201 cells with independent cDNA preparations of WT or F747S α1-subunits (two per construct; with identical β3, α2δ1 and EGFP cDNAs). EGFP served as transfection marker. We first confirmed that the number of EGFP-positive cells (quantified using ImageJ in three pictures per dish; three dishes per construct per transfection) correlates with the GFP signal obtained in Western blots normalized to α-tubulin (data not shown). Then, Cav1.3 α1 band intensities were normalized to the GFP signal, i.e. number of transfected cells. Normalization to α-tubulin only was performed as described previously (13–15).

### Electrophysiological recordings in tsA-201 cells

For whole-cell patch-clamp recordings patch pipettes from borosilicate glass (cat# 64-0792, Warner Instruments, Hamden, CT, USA) were pulled in micropipette puller (Sutter Instruments, Novaton, CA, USA) and fire-polished using an MF-830 microforge (Narishige, Tokyo, Japan) with a final resistance of 2.0–5.0 MΩ. Recording solutions contained (in mm): *bath solution*: 15 CaCl_2_, 10 HEPES, 150 Choline-Cl, 1 MgCl_2_, adjusted to pH 7.3 with CsOH; *pipette solution*: 135 CsCl, 10 Cs-EGTA, 1 MgCl_2_, 10 HEPES, 4 Na_2_ATP, adjusted to pH 7.3 with CsOH. Recordings were performed on room temperature (20–24°C) using the Axopatch 200B amplifier (Axon Instruments) and digitized (Digi-data, 1322A digitizer, Molecular Devices, San José, CA, USA) at 50 kHz, low-pass filtered at 1–5 kHz and compensated for 60–99% of the series resistance and subsequently analyzed using pClamp 10.7 software (Molecular Devices, San José, CA, USA). All voltages were corrected for a liquid junction potential of −9.3 mV and leak subtraction was done online (P/4 protocol, activation curves, tail current protocol) or offline (5 s pulses, steady-state inactivation protocol, pharmacological experiments). The holding potential (HP) was set to −89 mV.

Current–voltage (*I*–*V*) relationships were obtained by 25 ms square pulse depolarizations from the holding potential to various test potentials (5 mV steps) and data were fitted to the following equation:

$$ I={G}_{\mathrm{max}}\ast \left(V-{V}_{\mathrm{rev}}\right)/\left(1+\exp \left(-\left(V-{V}_{0.5,\mathrm{act}}\right)/{k}_{\mathrm{act}}\right)\right) $$

where *V*_rev_ is the extrapolated reversal potential, *V* is the test potential, *G*_max_ is the maximum slope conductance, *V*_0.5,act_ is the voltage of half-maximal activation, and *k*_act_ is the activation slope factor.

The voltage-dependence of Ca^2+^ conductance was fitted according to a Boltzmann relationship:

$$ G={G}_{\mathrm{max}}/\left(1+\exp \left(-\left(V-{V}_{0.5,\mathrm{act}}\right)/{k}_{\mathrm{act}}\right)\right) $$

Inactivation kinetics of the channels were examined by pulsing cells to *V*_max_ (voltage of maximum activation) for 5 s from holding potential and quantified at pre-defined time points (50, 100, 250, 500, 1000 and 5000 ms) as the remaining current in % of the peak inward current.

Steady-state inactivation was determined by calculating the ratio between current amplitudes of a control versus a test pulse (*I*/*I*_control_; 20–75 ms to *V*_max_) separated by a 5 s conditioning step to various potentials (10 mV increments; 30 s between sweep starts; from holding potential) and plotted as a function of voltage. Steady-state inactivation curves were fitted using a modified Boltzmann equation:

$$ I={I}_{\mathrm{ni}}+\left(1-{I}_{\mathrm{ni}}\right)/\Big(1+\exp\ \left(\left(V-{V}_{0.5,\mathrm{inact}}\right)/{k}_{\mathrm{inact}}\right)+{I}_{\mathrm{ni}} $$

where *I*_ni_ is the non-inactivating current component, *V* is the membrane potential, *V*_0.5,inact_ is the voltage of half-maximal inactivation and *k*_inact_ is the slope factor.

Window currents were obtained by multiplying the fractional steady-state inactivation at a given voltage with the corresponding average current density (pA/pF) at the given potentials of the *I*–*V* relationships.

Channel deactivation (tail current kinetics) was determined by a 10 ms depolarization to the reversal potential followed by 20 ms long repolarizations to various test potentials (10 mV steps).

Recovery from inactivation was determined by 25 ms test pulses to *V*_max_ at different decreasing time-points (between 0.001 and 20 s) after a 2 s conditioning pulse to *V*_max_. Sweep-to-sweep interval was 30 s. Data were sampled at 10 kHz and filtered at 2 kHz.

For pharmacological analysis, cells were depolarized using a 100 ms square pulse to the *V*_max_ of the individual cell with the frequency of 0.1 Hz from the holding potential. Cells were continuously perfused by an air pressure-driven perfusion system (BPS-8 Value Control System, ALA Scientific Instruments) with bath solution in the presence or absence of isradipine (Sigma Aldrich, D8418) with the flow rate of ~350 μl/min. Isradipine stock solutions were prepared in DMSO and stored at −20°C. On each recording day drug dilutions (1:1000 in bath solution for test concentrations, and 1:100 for 3 μm concentration) were freshly prepared from respective isradipine stocks. On each experimental day, current rundown was measured in individual cells using the same perfusion chambers subsequently used for isradipine experiments but filled only with bath solution. Current rundown was linear and similar on different experimental days. Current rundown was fitted by linear regression and subtracted from drug inhibition data. Drug application (two test concentrations cumulatively, followed by full block using 3 μm isradipine) was started after at least three constant control sweeps during initial bath solution perfusion.

Concentration for half maximal inhibition (IC_50_) was determined by fitting the data according to the following equation:

$$ Y=\mathrm{Bottom}+\left(\mathrm{Top}-\mathrm{Bottom}\right)/\left(1+10\hat{\mkern6mu} \left(\left(X-{\mathrm{LogIC}}_{50}\right)\right)\right), $$

where *Y* is the percent current in the presence of drug and *X* the log of the drug concentration.

### Molecular modelling

Structures of the WT Cav1.3 α1-subunit and the F747S mutant were predicted in three different gating states, activated-open (voltage-sensors "up," activation gate open), inactivated (voltage-sensors "up," activation gate closed) and resting/deactivated (voltage-sensors "down," activation gate closed), by generating homology models based on the available high resolution cryo-electron microscopy (EM) structures of voltage-gated Ca^2+^- and Na^+^-channel pore-forming subunits. The following structures were used as templates: activated-open state: open-stabilized Nav1.5 α-subunit structure (PDB accession code: 7FBS) (47); inactivated state: Cav1.1 α1-subunit structure (PDB accession code: 5GJV) (41); resting state: NavAb disulfide crosslinked mutant α-subunit (PDB accession code: 6P6W) (46). Activation gate conformations were also compared with the recently published inactivated state cryo-EM structure of the Cav1.3 Ca^2+^ channel complex (PDB accession code: 7UHG) (11).

Homology modeling has been performed using Rosetta and MOE (Molecular Operating Environment, version 2020.09, Molecular Computing Group Inc., Montreal, Canada) (57). Additionally, ab initio Rosetta was used to generate structures for loops that were not resolved in the original templates (58). The structures for the F747S mutant were derived from the respective wild-type model by replacing the mutated residue followed by a local energy minimization using MOE. The C-terminal and N-terminal parts of each domain were capped with acetylamide (ACE) and N-methylamide to avoid perturbations by free charged functional groups. The structure model was embedded in a plasma membrane consisting of POPC (1-palmitoyl-2-oleoyl-sn-glycero-3-phosphocholine) and cholesterol in a 3:1 ratio, using the CHARMM-GUI Membrane Builder (59). Water molecules and 0.15 M KCl were included in the simulation box. Energy minimizations of WT and mutant structures in the membrane environment were performed. The topology was generated with the LEaP tool of the AmberTools20, using force fields for proteins and lipids, ff14SBonlysc and Lipid14, respectively (60). The wild-type and mutant structures were gradually heated from 0 to 300 K in two steps, keeping the lipids fixed, and then equilibrated over 1 ns. Then molecular dynamics simulations were performed for 100 ns, with time steps of 2 fs, at 300 K and in anisotropic pressure scaling conditions. Van der Waals and short-range electrostatic interactions were cut off at 10 Å, whereas long-range electrostatics were calculated by the Particle Mesh Ewald (PME) method. PyMOL was used to visualize the key interactions and point out differences in the wild-type and mutant structures (The PyMOL Molecular Graphics System, Version 2.0 Schrödinger, LLC).

### Statistics

Data were analyzed using Clampfit 10.7 (Axon Instruments), Microsoft Excel, SigmaPlot 12.2 and 14.5 (Systat Software, Inc) and GraphPad Prism 8 software (GraphPad software, Inc). All values are presented as means ± SEM for the indicated number of experiments (*n*) unless stated otherwise. Data were analyzed by unpaired Student’s *t*-test, one-way ANOVA followed by Bonferroni post hoc test, and *F*-test as indicated. Overall statistical significance was set at *P < 0*.05.

## Supplementary Material

Supplemental_Figure_1_ddac248

Supplemental_Figure_2_ddac248
