# Supplementary material for: Germline de novo variant F747S extends the phenotypic spectrum of CACNA1D Ca2+ channelopathies
Source: Hum Mol Genet. 2022 Oct 8;32(5):847–59. doi: 10.1093/hmg/ddac248 (PMC9941835; doi:10.1093/hmg/ddac248)
Supplement: Supplemental_Figure_1_ddac248 [file supplemental_figure_1_ddac248.pdf]

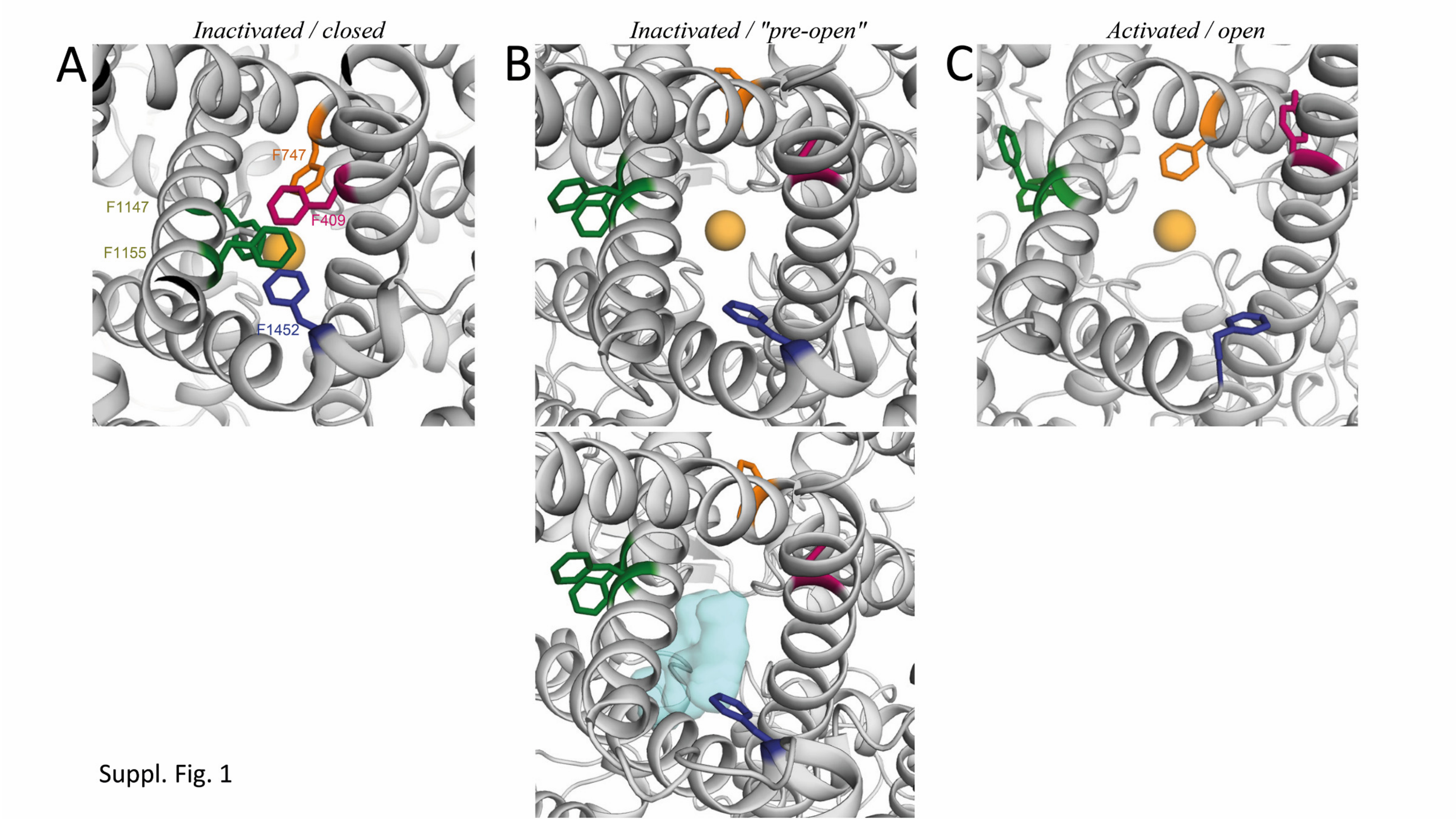

Supplemental Figure 1: Bottom view of the wild-type Cav1.3 activation gate in different conformations during the gating cycle

A. Bottom view of the activation gate formed by the cytoplasmic ends of the S6 transmembrane helices in a homology model of the inactivated closed conformation based on the cryo-EM structure of the Cav1.1 α1-subunit (voltage-sensors "up", PDB accession code: 5GJW). The S6 phenylalanines, which are responsible for sealing the gate, are colored

according to their respective repeat (repeat I - red, repeat II - orange, repeat III - green, repeat IV - blue). In the inactivated state they point into the gate and consequently prohibit the entrance of water. **B.** Model based on the recently published cryo-EM structure of the Cav1.3 α1 subunit (top). Although structurally analyzed under similar conditions (zero membrane voltage, no bound drug) in a putative inactivated state (voltage-sensors "up") the phenylalanines are turned outwards and therefore do not seal the gate anymore permitting water molecules / water wire (depicted as cyan surface) in the gate (B, bottom panel). An unsealed conformation with phenylalanines pointing away from the ion conducting pathway is also observed in the activated open conformation of the model (C).

C. An activated-open state homology model based on the Nav1.5 Cryo-EM structure (PDB accession code: 7FBS), shows in addition to the outward pointing phenylalanines, a widening of the gate by displacement of the S6 helices.

A Ca^{2+} ion is represented as an orange ball.
