# Supplementary material for: Germline de novo variant F747S extends the phenotypic spectrum of CACNA1D Ca2+ channelopathies
Source: Hum Mol Genet. 2022 Oct 8;32(5):847–59. doi: 10.1093/hmg/ddac248 (PMC9941835; doi:10.1093/hmg/ddac248)
Supplement: Supplemental_Figure_2_ddac248 [file supplemental_figure_2_ddac248.pdf]

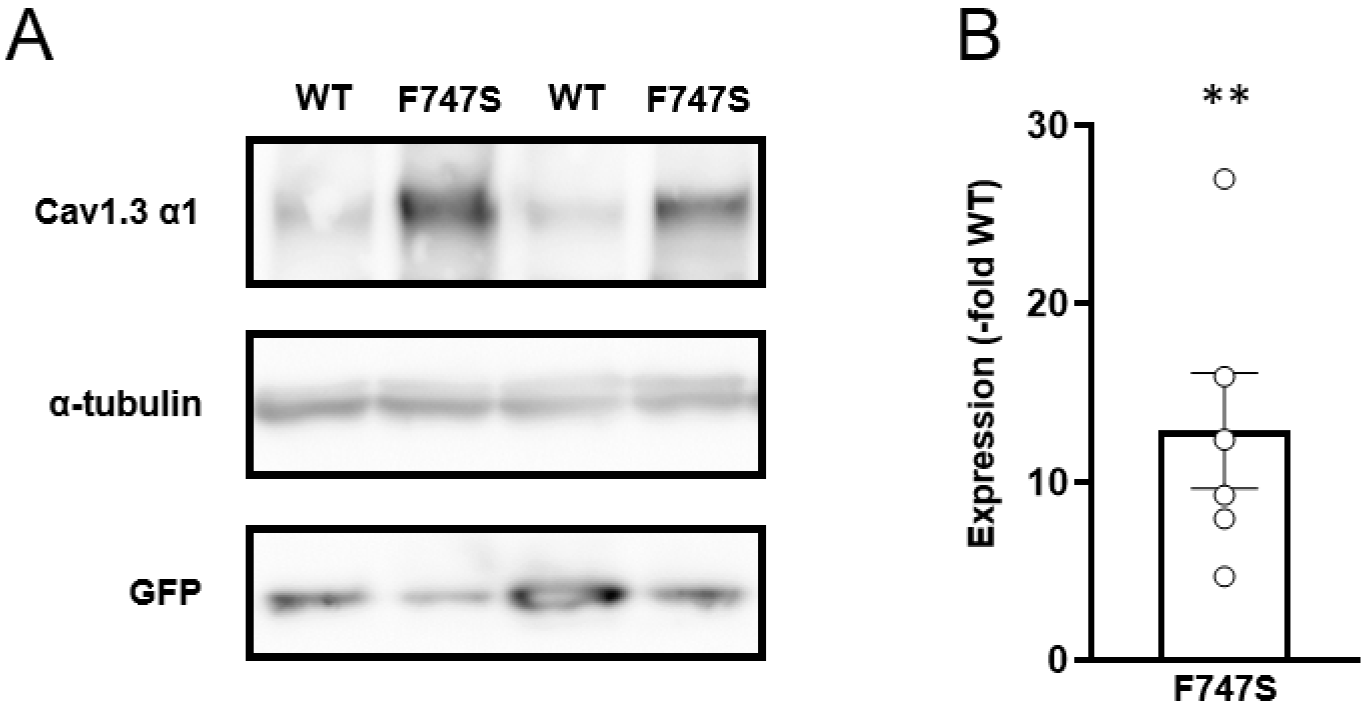

Suppl. Fig. 2

Supplemental Figure 2: Expression levels of total WT and F747S Cav1.3 α1-subunit protein in tsA 201 cells. Western Blot analysis of membrane preparations from tsA 201 cells transiently transfected with WT or F747S α1, β3, α2δ1 and GFP. GFP was used as transfection marker. A. Representative Western Blot of three independent experiments with two independent cDNA preparations of WT or F747S α1-subunits. B. Quantification of the relative protein expression levels. The α1 immunoreactivity signal was normalized to GFP (to account for variability in the transfection efficiency) and the protein level of F747S is given in comparion to WT samples (-fold) analysed on the same gel. Means ± SEM. Statistical significance was determined using one-sample Student's t-test ($p=0.014$) with WT expression level corresponding to 1.
